# Reverberation effect of communication in a public goods game

**DOI:** 10.1371/journal.pone.0281633

**Published:** 2023-02-27

**Authors:** Dmitri Bershadskyy

**Affiliations:** Department of Management and Economics, Otto-von-Guericke University, Magdeburg, Germany; Kyushu Daigaku, JAPAN

## Abstract

Using a public goods laboratory experiment, this paper analyzes the extent to which face-to-face communication keeps its efficiency gains even after its removal. This is important as communication in real world is costly (e.g. time). If the effect of communication is long-lasting, the number of communication periods could be minimized. This paper provides evidence that there is a lasting positive effect on contributions even after communication was removed. Yet, after the removal, the contributions are lower and abate over time to the previous magnitude. This is referred to as the reverberation effect of communication. As we do not observe an effect of endogenizing communication, the strongest driver of the size of the contributions is the existence of communication or its reverberation. Eventually, the experiment provides evidence for a strong end-game effect after communication was removed, insinuating communication does not protect from the end-game behavior. In total, the results of the paper imply, that the effects of communication are not permanent but communication should be repeated. Simultaneously, results indicate no need for permanent communication. Since communication is conducted using video-conference tools, we present results from a machine learning based analysis of facial expressions to predict contribution behavior on group level.

## Introduction

Most cooperation processes involve communication between partners. Communication is a cheap and simple means to increase cooperation. Several laboratory experiment support this observation [1–3]. However, only few studies focus on the long-run effects of communication [4–6]. Yet, this topic is important since even behavioral measures (e.g. providing communication) induce costs [7] which should be minimized. If the changes induced by communication are permanent, communication can be removed to save money and, in broad terms, to avoid the impression of persistent paternalism. Thus, it is the central focus of this article to investigate how persistent are the efficiency gains caused by communication in a public goods experiment.

In so doing, this article contributes to the literature by combining the general structure in [3] (i.e. public goods experiment with communication) and the experiments on public goods with restarts with strangers in [8–10]. The goal is to assess whether the long-term effects of communication are powerful enough to spill over to new groups that did not have a chance to communicate.

In general, the insufficient provision of public goods was experimentally shown using the voluntary contribution mechanism (VCM) in various setups [11, 12]. In a public goods experiment, we used information and communication technology (ICT) to induce face-to-face communication to analyze whether the positive effects wear off over time. Note that often literature discusses face-to-face communication as one where all subjects are in the same room. In contrast we applied an ICT where all group members can see each other and talk. Still, we refer to it as face-to-face as prior experiments indicated that at least for the public goods game there are no differences whether subjects discuss it face-to-face in person or face-to-face via software [3]. However, using the ICT enables us to record and analyze communication as is presented later. In the context of the paper, the contribution behavior after removing communication is addressed as its reverberation effect. To investigate this effect, we focus on two questions.

First, do efficiency gains of a behavioral intervention prevail after its removal? In a broader sense this questions how past outcomes influence future contribution behavior. Previous research often focused on the effects of changing the “good” institutional environment to a “bad” one and vice versa. This paper adds the focus on endogenizing such a change. In some treatments, subjects have to finance communication first. This constitutes a second-order public goods dilemma. Therefore, the second question is, whether failing or succeeding to finance communication influences future contribution behavior. This is of interest as failing to fund an efficiency enhancing intervention can lead to signals in both directions which is discussed in chapter two.

The experiment consists of three blocks of ten periods each where subjects meet in different groups of four people in every block. The results indicate that the combination of a standard VCM in block one and a VCM with pre-play communication (C-VCM) in block two yields three major findings for subjects without communication in block three. First, with the exemption of the last period of block three, the contributions are higher and more stable. Second, the end-game effect is more severe, as the contributions decrease very strongly in the last period of the block. Thus, the paper provides experimental evidence that there is a positive reverberation effect of communication after its removal. However, the gains are abating. Third, we do not find sufficient evidence for any type of signaling. Further, we can analyze communication files concerning content and facial expression. For the latter, we developed a novel automatic approach trained specifically on this data set and technically described in [13] to predict defection in the group. Results indicate prediction rates significantly better than random guesses.

The outline of the paper is as follows. After providing an overview of relevant literature in section 2, the paper illustrates the experimental setup in section 3. The presentation and discussion of the main results take place in section 4 and 5. Section 6 provides the conclusion.

### Literature

The classical findings in the experimental literature on VCM are an inefficient provision of the public good and a downward trend of individual contributions over time [11, 12]. In the context of this paper communication is a behavioral intervention raising the contributions to the public good. Thus, we briefly discuss (i) canonical findings in environments without any intervention, (ii) different types of interventions, and (iii) specific effects of communication. Subsequently, the literature review will address the topics of (iv) path dependence (prior experiences) and (v) end-game behavior.

First, the canonical result for VCM without any intervention is that the contributions to the public good are below the social optimum and decrease over time. A large share of subjects behaves as conditional cooperators, i.e. people who provide more to the public good when other members of the group contribute a lot [14, 15]. Furthermore, contributions decrease strongly in the last periods, which is referred to as the end-game effect [8, 16].

Second, to increase contributions, different types of interventions can be applied. In laboratory experiments, this includes introducing punishments or rewards in the VCM. These interventions simulate formal institutions in the laboratory. [17, 18] illustrated how positive (negative) taxes for selfish (non-selfish) players lead to higher contribution rates. [19] showed how competition between sanctioning and non-sanctioning institutions led to individuals choosing the sanctioning one. [20] illustrated that subjects with a choice between a sanctioning and a rewarding institution, preferred the rewarding one despite it being inferior. A different experimental approach leading to an increase of contributions without formal institutions are restarts [8–10]. After completing a VCM of several rounds the VCM was simply restarted. The contributions of the first period after the restart were higher than in the final periods before restart yet lower than in the first period before restart. Unlike, [21] argued that this restart effect does not occur when the subjects are replaced periodically by new members. Further, [22] argues that the restart effect is the stronger if combined with communication.

Third, in contrast to restarts, another informal measure has a more fundamental effect. [6] showed that face-to-face communication increased contribution rates in public goods experiments to approximately 100%. Following these results research focused on the reasons for the increase. [23] distinguished between E-Mail communication and face-to-face communication, indicating that E-Mails increased cooperation but less than face-to-face communication. [1] illustrated that face-to-face communication increases efficiency independent of the ability to monitor prior contribution quality. [3] extended the findings by distinguishing between more types of communication in a public goods experiment. The authors illustrated that it is the combination of verbal and audio-visual communication that enhances efficiency the most. Audio communication, passive communication, or visual identification without verbal communication did not achieve the contribution rates of in-person face-to-face communication, or video conference treatments. The difference between face-to-face communication and video conference was negligible. Likewise, [2] compared different types of communication and found a strong effect of face-to-face communication. In an early overview, [24] concluded that face-to-face communication, including video conferences, is an effective tool to increase efficiency in different types of experiments and is important for real-world applications. These findings are supported by a more recent literature overview [25]. Despite a large research body on communication in economic experiments, one issue appears only rarely—the persistence of the communication effect. [6] argues that contribution rates decrease from group optimal levels after communication has ended. [5] provides evidence of a limited long-run effect of communication in a Bertrand oligopoly setup. [4] studies long-run effect of communication on conflict resolution using a Tullock contest and shows a strong and persistent effect of communication. Yet, these studies do not examine the possibility of subjects changing groups, i.e., ending up in a new group that did not communicate yet. However, such changes are exactly what occurs in the real world, which is the reason to investigate whether communication effects spill over to completely new groups as is the aim in *hypothesis one* of this article.

Most of the aforementioned experiments analyzing face-to-face communication focused on costless communication. Yet, this is not true in the real world. Early research [26–28] depicted that the positive effect of communication persisted even when communication became costly. However, this should be considered with caution as the use of communication devices decreases when communication is not for free [29]. Still, the question, what happens when communication in a VCM gets rejected after people experienced its efficiency, remains open. This refers to the effect of previous institutional experience (e.g. path dependence and signaling).

Fourth, experimental evidence on path dependence is mixed and uses different environments. [30, 31] illustrated that coordination failure in minimum-effort games can be resolved by changes in financial incentives even without changing the equilibrium outcomes, implying that experience there has no decisive effect on future behavior. In a platform competition experiment, [32] tested the QWERTY phenomenon illustrating that subjects switch to a more efficient platform. Thus, the threat to get caught in a bad equilibrium as originally described in [33] did not find experimental support.

In contrast to these studies, there is evidence for path dependence and that groups can fail to adapt perfectly to a changing environment. [34, 35] traced this back to incomplete information. According to [36], path dependence arose when the preferences of subjects changed gradually yet separately. [37] argued that even under complete information, a commonly known change in an institution fails to affect the expectations of the subjects and therefore their behavior. Further, [38] investigated the differences in spillover effects between nudges and push measures in different Ultimatum and Prisoner’s Dilemma Games. [39] demonstrated the spillover effects of an efficiency-providing institution on a simultaneously existing inefficient institution. [40] indicated lasting spillover effects of a sanctioning institution providing leniency to whistleblowers even after the removal. [41] illustrated how being exposed to more cooperative environments in a repeated prisoner’s dilemma led individuals to become more prosocial and punish selfish behavior in a subsequent one-shot game. Finally, [42] implemented a related public goods experiment, yet with only two blocks. The authors analyze cooperation rates where the initially high financial incentives to cooperate were reduced afterward. The results indicate that after removing high incentives, cooperation deteriorates and may become smaller than in the non-treated group. Summing up, while evidence of path dependence is not completely unambiguous, the majority of research indicates that prior institutional forms influence the behavior after an institutional change.

Further, choice on prior institution can send out a signal to other group members. Providing high contributions to the second-order public good can signal trust for contributions in the first order public good. This would be in line with standard models on conditional cooperation in VCM [14, 43]. Yet, models on the signal value of trust indicate a possible second direction of this signaling effect. Low trust in cooperation may increase the demand for establishing institutions as in the model in [44]. Providing an efficiency enhancing institution can therefore signal an environment with a lot of selfish agents and may result in defection of conformist agents [45]. Thus, it is a priori unclear whether low contributions to communication would be a positive signal (i.e. there is no need to spend additional money on it) or a negative one (not funding the institution signals low level cooperation for the upcoming VCM). This leads to *hypothesis two*.

Finally, we address the role of end-game behavior in public goods experiments and the role of communication in it. In the final period, there are no expected benefits from longstanding cooperation which leads subjects to contribute at rates closer to the Nash Equilibrium. Further, the end-game effect is robust concerning different designs, e.g. non-definite time horizons in [46] and sequential contributions in [47]. Moreover, it seems that communication in public goods experiments does not solve it [3]. For our experiment, this implies that despite randomization, subjects could start learning end-game behavior. [16] introduced a learning theory for end-game behavior in repeated finite Prisoner’s Dilemma games. According to it, individuals learn when other players start defecting from the socially optimal behavior and try to anticipate it in the next repetition. Yet, until now there is no experimental evidence on such an end-game learning behavior in experimental setups with communication. This shall be investigated using *hypothesis three*.

The discussed literature leads to three hypotheses that are tackled by the experimental design.

*Hypothesis 1 (Reverberation effect hypothesis)*
  Experiencing the efficiency gains of C-VCM as compared to VCM yields subjects to contribute more to the public good in a future VCM despite reshuffling members of the groups.
*Hypothesis 2 (Signaling effect hypothesis)*
  Failing to build an efficiency providing intervention is a signal that affects later contributions within the group.
*Hypothesis 3 (End-game hypothesis)*
  Experiencing the end-game in the first two blocks leads to a strong end-game effect in block three without communication.

In extension to the classical analysis, we further present the results of communication analysis in Section 4.4. Please note that these do not underly any causal relationships and are purely exploratory.

### Experimental design

The design of the total experiment consists of three blocks. In the beginning, the individuals are informed that only one of the three blocks will be cash-effective. Therefore, we avoid income accumulation between the blocks. Furthermore, the design includes randomization between the blocks. Following a round-robin design, it is ensured that subjects cannot encounter each other after they were members of one group before. This information is public.

**Fig 1:**
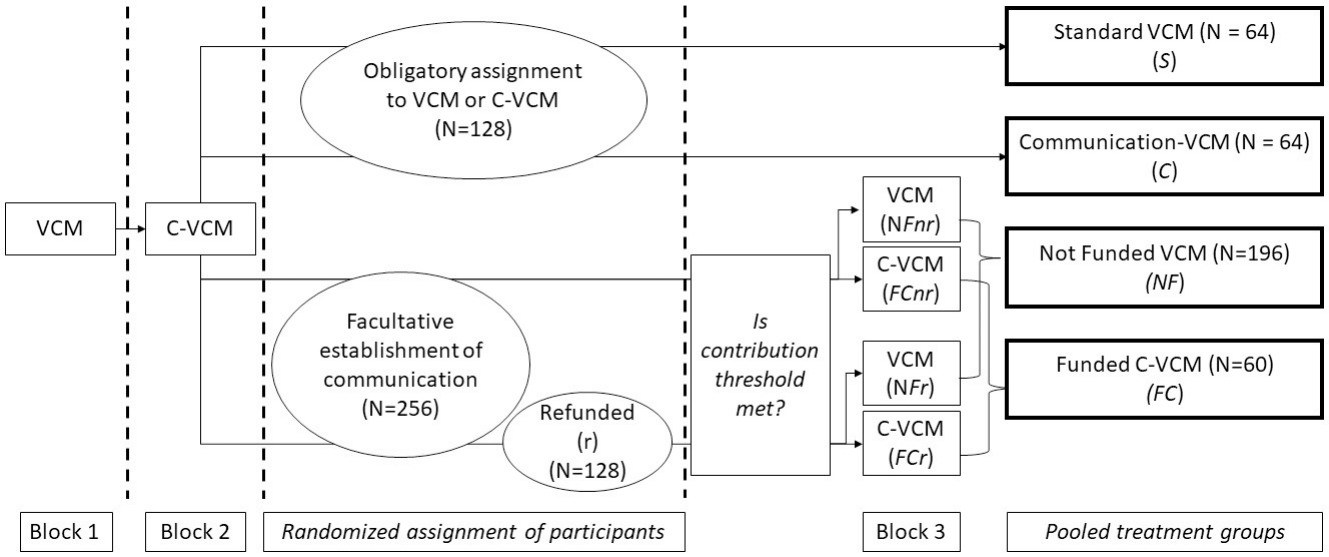
Experimental design. VCM: voluntary contribution mechanism; C-VCM: voluntary contribution mechanism with pre-play communication; No refund (nr): Individuals pay their investment independent of whether the threshold was met; Refund (r): Individuals pay their investment only if the threshold was met. N depicts the number of subjects.

The block specific instructions were distributed and read aloud by the instructor prior to every block. The instructions are listed in S1 File. The core of every block is the standard VCM which is kept unchanged in every block for all subjects. The pay-off function of individual *j* in period *k* is defined as:

$$\pi_{\text{jk}}\left({\text{g}}_{\text{jk}}\right)=\;{\text{z-g}}_{\text{jk}}+\frac{\alpha }{\text{n}}\overset{n}{\underset{\text{j}=1}{\sum }}{\text{g}}_{\text{jk}},\;\text{j}=1,\ldots 4$$

with the initial endowment (z) = 20 Laboratory Dollar (LD), the efficiency multiplier (α) = 2, g_jk_ representing the amount of LD subject j invested in period k. In every block, subjects repeat this VCM for 10 periods in constant groups of four individuals. After every period, subjects are informed about their payoff and the contributions of other group members. After the last period of the block, individuals receive information about their total payoff for this block. Block one includes only the standard VCM. In block two there is three-minute long pre-play communication (C-VCM). All subjects play block one and block two. Treatments differ only concerning block three. One-sixth of the subjects repeat the VCM (as in block one). Another sixth of subjects repeat the C-VCM (as in block two). These subjects work as control groups. The remaining two-thirds of the subjects have to meet a financial threshold to jointly finance communication in block three as a group. The costs of the communication platform (public information) were in total 32 LD for a group of four individuals. Please note that if everybody followed the Nash Equilibrium strategy they would receive 200 LD and if everybody followed the socially optimal strategy then 400 LD. The factual average benefit of communication for every subject was approximately 64 LD. Thus, the average benefit of communication was on average eight times higher than the average costs. To finance the communication platform, subjects have to indicate their respective contributions. Here we distinguish two different payment options: Half of the subjects receive their money back if their group misses the threshold value for the communication platform (refund). The other half lose their invested money if the groups fails to finance communication (no refund). Investigating the different refund options aims to address the ambiguous ways contributions to the second-order public good can be seen for the VCM, since the refund option reduces the risk of losing money. Groups that achieve the threshold value proceed with the C-VCM and others proceed with the standard VCM. After the end of the third block, the subjects are informed which block became cash-effective and answer a questionnaire. The complete experimental structure is depicted in Fig 1.

This design allows a within subject analysis of the reverberation effect, i.e. how the experience of efficiency gains between blocks one and two influences subjects’ behavior in block three. Further, the design enables a between subject analysis of potential signaling effects concerning the funding of communication, i.e. differences of contributions in groups where funding of communication was successful vs. groups where it was unsuccessful.

In total, the paper distinguishes two major treatments and two endogenously formed groups (see Table 1). The standard treatment (S) is composed of subjects that simply repeated VCM in block three. In the communication treatment (C) subjects repeated the C-VCM procedure. The Not Funded group (NF) consists of subjects who had the chance to fund the communication platform but whose group missed the threshold. The subjects stem from both payment options (Refund and No Refund). Likewise, in the group Funded Communication (FC) there are all subjects who met the financial threshold to continue with C-VCM in block three independent of the refund option. The explanation for pooling the refund and non-refund options is provided in the result section. As will be shown in the results section the two different payments schemes (Refund vs. no-Refund) do not influence average contribution behavior. Please note, that the results would not change if regarded separately.

**Table 1:** Overview of groups.

	Communication		No Communication	
Funded with no Refund	FCnr	} **FC**	NFnr	**} NF**
Funded with Refund	FCr		NFr	
Exogenous Provision	**C**		**S**	

The subjects were recruited from the subject pool of the Magdeburg Experimental Laboratory of Economic Research (MaXLab) and consisted of students from the Otto-von-Guericke University Magdeburg (Germany). In total 384 students took part in the experiment. Due to power analysis based on [3] no treatment should have less than 24 subjects to detect communication effect on group level. Since other effects, were likely to be smaller, the actual number of subjects was higher. The duration of the experiments in total was between 70 and 90 minutes. After the end of the experiment, the payoff of one of the blocks was converted to euro (1 Laboratory Dollar = 4.5 Cents). The average payoff was around 16 €. The experimental design was executed in z-Tree [48]. The experiment was organized using hroot [49]. Written informed consent was obtained by all subjects. The study did not require an IRB review by German law because it only uses standard experimental protocols in line with the German Association of Experimental Economists (GfeW).

## Results

The result section is divided into different types of analysis. First, we present descriptive statistics. Second, the paper examines whether having the option to finance communication matters. Third, the paper focuses on the effects of removing communication. Fourth, we analyze communication protocols and illustrate whether non-verbal communication can be used to predict certain defection behavior in groups.

### Descriptive statistics

**Fig 2:**
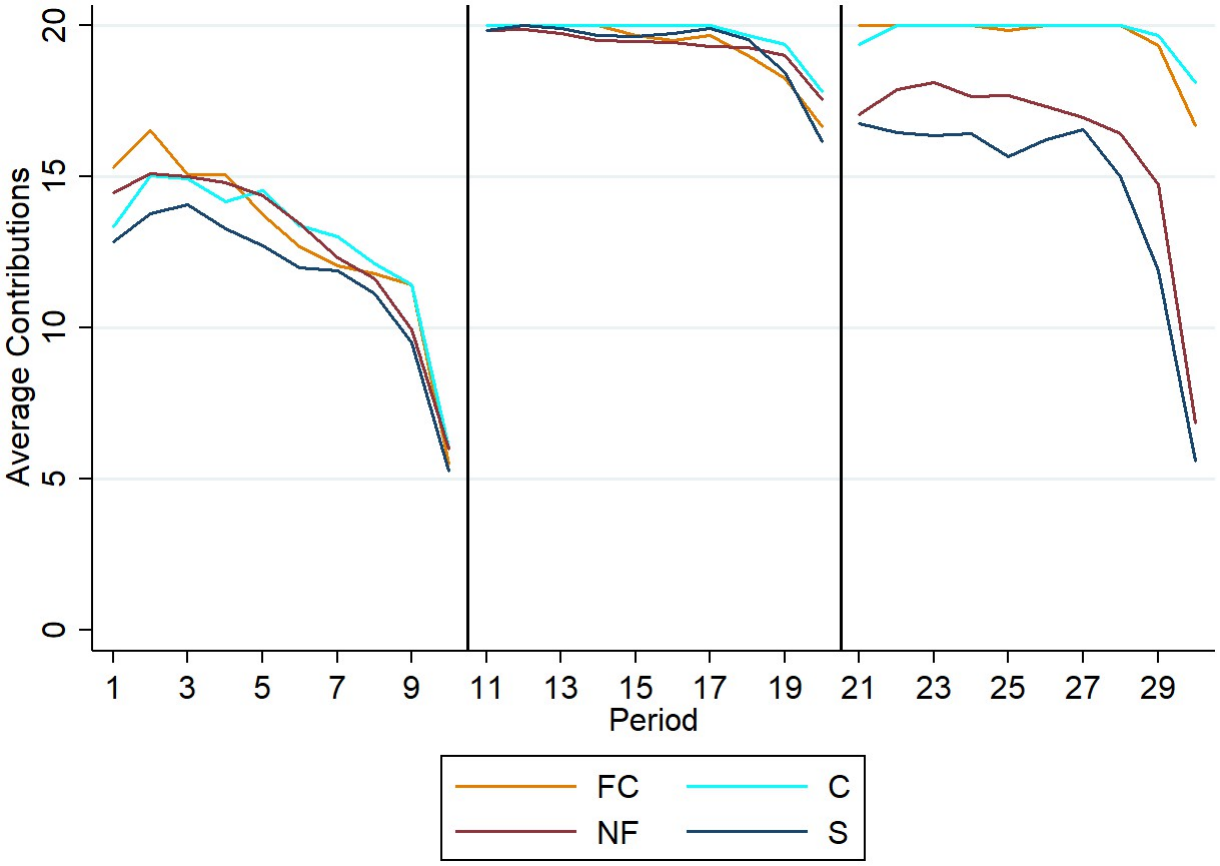
Average contribution rates in all blocks for respective treatments. Periods 1–10 constitute block one, 11–20 block two, and 21–30 block three respectively.

Fig 2 sums up general contribution behavior throughout all blocks of the experiment. In block one we observe the typical decline of contributions over the ten periods. In block two there are high and stable contribution rates. Thus, results from [3] are reproduced. In block three, where the actual treatment differences matter, we observe two major findings. First, there are differences between subjects with communication (FC and C) and without (S and NF). Note, that contributions in NF are higher than in S. Yet, the differences are not significant and could possibly originate from minor differences in samples in these two groups as subjects in NF had higher contributions in block one, as well. Second, the contribution behavior of subjects without communication in block three is potentially different as compared to block one. We further provide some basic information about the sample of subjects in Table 2 while stressing the difference between endogenous groups NF/FC and the actual treatments nR and R.

**Table 2:** Sample pool statistics.

	Block 3	Block 3	Block 3	Block 3
	no Refund (nR)	Refund (R)	S	C
Sessions	8	8	4	4
Total Subjects	128	128	64	64
Share Male	51.56%	60.93%	56.25%	50.0%
Average age	23.96	24.10	23.28	22.92

### Signaling effect

To examine the effects of having the option to finance communication, the analysis focuses on the behavior of subjects in block three in all treatments. Table 3 and Fig 2 indicate significant differences between subjects with communication in block three and those without. However, to analyze the value of choice, it is necessary to compare the treatments where subjects had a choice with those where they were exogenously assigned to a VCM or C-VCM. This means comparing groups FC vs. C (with the communication) and NF vs. S (without communication). The results from two-sided Mann-Whitney (MW) tests, conducted on the aggregates of all ten periods on the group level of contributions, are presented in Table 3. Therefore, there were no significant differences between groups with opportunity to finance communication by meeting a financial threshold and those without. This is true for repeating VCM (p = 0.2142) and repeating C-VCM (p = 0.2966). However, in total, groups with communication (C-VCM) had significantly (p<0.001) higher contributions than groups that without (VCM).

**Table 3:** Differences of contributions in block three aggregated on the group level over 10 periods and p-values of the MW-test.

	NF	S	FC	C	NF+S	FC+C
Mean	642.898	587.750	783.467	788.750	629.323	786.194
Observations	49	16	15	16	65	31
MW-test	0.2142		0.2966		0.0000	

This result has two implications. Firstly, giving subjects the option to finance the platform did not influence their behavior. Secondly, we can pool the two groups for the following analysis. Further, it is important to stress that the two different payment schemes (refund and no refund) neither had an effect on the overall establishment of communication in block three nor did it generate differences in the contribution behavior in the third block as a whole. S1 Table illustrates that neither in the case of a successfully nor unsuccessfully funded communication did the type of choice have a significant effect.

The only significant difference can be observed when analyzing the first contributions after the unsuccessful formation of the communication platform. Hereby, there may be a certain mistrust effect. Individuals, who largely contributed to the communication platform but whose group did not meet the threshold, contributed less to the public good in the first period of block three. These results are displayed in S2 Table. However, the differences are not robust and disappear after the first period. Thus, we do not observe enough evidence to regard these groups as different and pool the data instead. Summing up this section, we do not observe major signaling effects. Instead, block three yields the observation that the most important criterion is whether communication took place.

### The reverberation effect

To examine the reverberation effect of communication after its removal, the contributions of subjects in block three are compared to blocks one and two, respectively. The subjects can be divided into two groups—with or without communication in block three. In short, Table 4 summarizes that individuals who did not have communication in block three contributed differently than in block two (p<0.001). In comparison, subjects who had communication in block three showed almost the same behavior as in block two (p = 0.4301).

**Table 4:** Differences in contributions between second and third blocks.

	Block 2	Block 3	Block 2	Block 3
	NF+S	NF+S	FC+C	FC+C
Average	767.2269	629.3231	781.0081	786.1935
Observations	65	65	31	31
Mann-Whitney	0.0000		0.4301	

The maximum value a group can contribute over 10 periods is 800 LD.

Analyzing the repetition of the standard VCM (see Fig 2 and Table 5), there are two major observations. Firstly, the initial contributions are higher in block three than in block one. This observation contrasts findings from laboratory experiments where the subjects simply repeated the VCM as in e.g., [8–10, 50, 51]. There, first contributions of block two were higher than the last contributions of block one but remained lower than the initial contributions in block one. Further literature that made use of such restarts to investigate the role of strategy by distinguishing between strangers and partners as in e.g., [43, 52] came to the same observations as stated in all prominent literature reviews on VCM [11, 12]: contributions decrease over time—despite restart effect. Still, in our setup the contributions in block three were significantly higher (630 LD) than in the first block (493 LD).

**Table 5:** Differences in contributions between first and third blocks.

	Block 1	Block 3	Block 1	Block 3
	NF+S	NF+S	FC+C	FC+C
Average	493.9615	629.3231	500.6774	786.1935
Observations	65	65	31	31
Mann-Whitney	0.0000		0.0000	

The maximum value a group can contribute over 10 periods is 800 LD.

This implies that the experiences in the second block (C-VCM), though being technically independent of block one and three, induced positive spillover effects. Simultaneously, the contributions do not achieve similar rates as in the C-VCM itself (see Table 4). This is important as it shows that the experience of the efficient C-VCM is not sufficient to induce equally strong long-term efficient behavior. Secondly, another notable difference between the two standard VCMs in blocks one and three is that in the former the contributions follow a steady decrease over time until there is a sharp decrease towards the end-game phase. In the latter, the contributions in the first seven periods are more stable and remain comparably high. However, in the end, there is no difference between contributions in the last periods of blocks one and three. Thus, the end-game behavior is much more severe in block three than in block one (Fig 2). This is striking as by design the individuals do not know which block will be paid out and are incentivized to perform in the best possible manner in every block respectively. This means that approaching the end of the total experiment is not a good explanation for such a sharp decrease in contributions. Instead, we consider it plausible that subjects experienced the end-game twice (in block one and two) and in accordance with the model in [16] anticipated the decline in the last round and thus, increased its severity.

The results are further supported using two dynamic panel Tobit regression models with random effects. The specification is based on the findings of [53] who stress its benefits and the fact that no other practically feasible estimator outperforms this specification. For robustness, we introduce a group-level version of the estimator. The individual-level model is represented by:

$$Contribution_{it}=\beta_{0}+\;\beta_{1}AvContr_{-it-1}+\beta_{2}t+\;\beta_{3}Block+\;\beta_{4}Block*t+\alpha_{i}+u_{i}+\;\varepsilon_{it}$$

with Contribution_it_ being the contributions individual i provided at period t (1–10), AvContr_-it-1_ the average contributions of the other three players in the group at the prior period, Block the respective block (1–3) which determines whether there was communication, α_i_ several control variables on the individual level. The limits of the model are at 0 and 20 LD. The group level model (with limits at 0 and 80 LD) is similar, yet leaves out the average contributions of the other members, since they are incorporated in the group contribution variable and uses control variables α_j_ on the level of the group j.


$$GroupContribution_{jt}=\beta_{0}+\;\beta_{1}t+\;\beta_{2}Block+\beta_{3}Block*t+\alpha_{j}+u_{j}+\;\varepsilon_{jt}$$

Consequently, the results from the regressions are depicted in S3 Table. The results support the observation that contributions in block three were significantly higher than in block one. They further indicate that the contributions decrease over time. To sum up, communication has a positive effect in block three. However, this effect is not stable but wears off over time towards the contribution rates obtained in the end-game of block one. Therefore, contributions achieved through communication in block two reverberate towards the levels achieved before.

Turning the focus on the repetition of the C-VCM in the third block implies the question of whether the subjects were learning from repeating the C-VCM. Since there is no previous literature on repeating C-VCM no well-grounded hypothesis was a priori possible. However, since the contributions in block two are on average at 96.46% there is almost no room for improvement. The regression results with the same regression model as before are depicted in S4 Table. Further, the analysis of average contributions does not yield significant results. The contributions in block three appear to be slightly higher and more stable for a longer period. However, the end-game effect is equally present in the repetition of the C-VCM and thus, in the end, the contributions declined to the same levels as in block two.

### Analysis of communication

After the presentation of main findings of the experiment, this section presents the exploratory analysis of the face-to-face communication that has been recorded. The results displayed in this section are a summary of the preprint [54] and the peer reviewed technical explanation of the approach to automatically analyze this specific set of video data [13]. We focus on two different categories: (i) content and (ii) facial expressions. For clarity, it has to be noted that for technical reasons it was neither possible to combine both categories i.e. analyze facial expression when a certain content was discussed nor to analyze the intonation of the voices.

**Fig 3:**
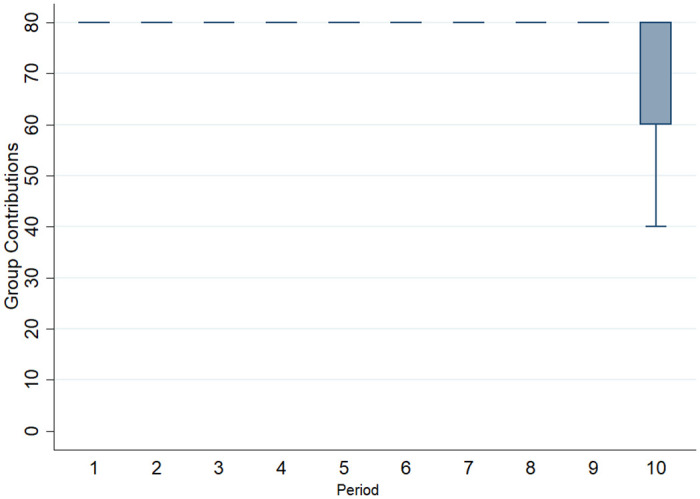
Boxplot of group contributions over ten periods (outliers not displayed). Note, the differences to Fig 2. The deviation from full contributions occurred in a minority of groups. Yet, these deviations were severe enough to decrease the overall contribution averages in a way displayed in Fig 2. Leaving out the outliers serves to highlight the lack of variance in periods 1–9.

As every communication consists of four subjects the analysis can only be performed at the group level. This means that we obtain 96 communication protocols from block two and 31 from block three (i.e., a total of 127 communication protocols). Further, due to the previously discussed strong effect of communication, the level of variance in contributions was low, as is shown in Fig 3. Therefore, it is only possible to analyze the effects of communication to the final period of the respective block. Despite this complicated initial position, several observations can be made.

#### Analysis of content

To analyze the content, communication videos were first transcribed. We did not apply the automatic methods described in [55] since the data structure was fundamentally different. Instead, using a codebook, two blinded coders conducted the classification displayed in Table 6. The classification is related to the one conducted in [3]. The two coders achieved a high level of interrater agreement ranging between 87% and 100% for the respective variables. For a more detailed depiction of different types of interrater agreements we refer to S5 Table. Noteworthy, the variable with the weakest agreement is End-game awareness. Yet, this variable is of special interest, because as was illustrated in Fig 3, only in the last period did the groups differ in their contribution behavior. In contrast to some variables that were easy to classify, the discussion of the end-game was sometimes ambiguous. For example, some groups mentioned terms like “last period” while others discussed the likelihood of decreasing contributions in the course of the block. This is a likely reason for the differences between coders. This leads to the question of how to proceed with data where coders did not agree. First, you can use only data where both coders agree. Second, you can add up the binary votes and consider these as values for how salient the statement was. The first approach leads to different sample sizes for different variables. Thus, the analysis focuses on the second approach. We further extend the list of variables by an individual and group level word count.

**Table 6:** Definition of the coded variables.

Variable	Definition	Coding
Full Investment	The participant(s) mentioned to invest full contributions	“0”—no, “1”—yes
End-game awareness	The participant(s) mentioned that they should contribute fully until/in the end. (No explicit agreement required)	“0”—no, “1”—yes
Previous experiences	The participant(s) discussed experiences from previous block or prior experiments	“0”—no, “1”—yes
Threats and Consequences	The participant(s) “threatened” potential free-riders by explaining consequences, e.g. they will reduce their contributions.	“0”—no, “1”—yes
Disagreement	How many players (temporarily) disagreed with the optimal solution after it was mentioned	Numbers from 1 to 4
Information provider	Which player in the team explained the dilemma/solution of the dilemma (first).	Numbers 1 to 16 (linked to the specific individual in every session)

As it is a priori unclear whether subjects change the content of their discussions from block two to block three, we will divide the analysis. In the following, we will refer to the 96 protocols from block two as First Time Communication (FTC) and 31 protocols from block three as Second Time Communication (STC). Table 7 summarizes descriptive statistics for both.

**Table 7:** Descriptive statistics of content variables and meta parameters for FTC and STC.

Variable	First Time Communication					Second Time Communication				
	Obs	Mean	SD	Min	Max	Obs	Mean	SD	Min	Max
End Game	96	.323	.435	0	1	31	.387	.460	0	1
Invest All	96	.979	.144	0	1	31	1	0	1	1
Disagree- ment	96	.083	.268	0	1	31	.097	.301	0	1
Prev. Expe-rience	96	.411	.459	0	1	31	.774	.405	0	1
Threats/ Conse-quences	96	.281	.422	0	1	31	.355	.469	0	1
Total word count	96	244.760	118.015	33	516	31	260.710	133.283	18	470
Ind. word count	382	61.521	55.108	0	306	31	65.715	62.004	1	237

The values for content parameters stem from two coders and are normalized to 1. Thus, the mean value denotes the percentage of cases in which the respective variable was identified by coders. We provide the observation numbers on group level in all categories but the last one (individual word count). There, observations are coded on the individual level.

To analyze the influence of the variables on contributions in the last period, we apply Tobit regressions (lower bound: 0 and upper bound: 80) for FTC and STC with three different regression models. In the simple regression (1 & 4), there is only the variable word count. In the next regression model (2 & 5), we add demographics on the group level (age, gender, study faculty). We note that including faculty follows the idea that students in economics are more likely to know the public goods game from their curriculum. Thus, they could theoretically explain the dilemma. However, the idea did not find empirical support as is illustrated in S6 Table.

In regression models (3 & 6), we add the classified variables (Invest All, End-game, etc.). The results are depicted in Table 8 and indicate that the total number of spoken words is linked to contributions, yet only for FTC. Further, we note that discussing the End-game significantly increases contributions to FTC and STC.

**Table 8:** Tobit regressions of contributions on group level for FTC and STC.

	FTC			STC		
	(1)	(2)	(3)	(4)	(5)	(6)
Total word count	0.160** (0.064)	0.167** (0.064)	0.205*** (0.071)	0.025 (0.083)	0.028 (0.082)	-0.171 (0.101)
Number of economists		4.827 (7.242)	0.326 (6.478)		2.341 (14.372)	3.366 (13.182)
Number of males		-3.243 (6.593)	-5.477 (6.137)		-6.036 (11.332)	-16.140 (11.513)
Aggregate of age		-0.506 (0.976)	-0.745 (0.869)		3.289* (1.893)	2.879 (1.706)
End-game			17.042** (8.400)			43.374** (18.523)
Invest All			34.902* (17.841)			(omitted)
Subjects against			-16.692 (11.040)			8.842 (21.194)
Previous Experience			-12.475 (7.653)			16.947 (11.651)
Threats and consequences			-6.736 (7.967)			28.534 (17.839)
Constant	63.492*** (15.054)	107.305 (89.452)	70.092 (82.141)	95.985*** (24.448)	-202.146 (171.12)	-164.268 (151.935)
Number of Observations	96	96	96	31	31	31

Standard error is denoted in brackets.

***/**/* denote significance of the coefficients at 0.01/0.05/0.1 levels respectively.

The variable *Invest All* was omitted in STC due to a lack of variation. See Table 7 for descriptive statistics.

The results so far focused on the group level. However, some of the gathered information is only plausible on an individual level, e.g. individual word count or information on who explained the dilemma. To have a comparable approach, we coded a dummy variable labeling the player who spoke the most in the group (i.e. talker). This enables the analysis of players who spoke the most words and those who provided information concerning differences in gender or faculty using Chi2-tests. The results indicate no significant differences between students concerning faculty, yet significant differences concerning gender. In block two (three) 73 out of 98 (25 of 31) individuals who talked the most were male. The results are significant at p<0.001 (p = 0.001). Concerning information providers, the results are similar. In block two (three) 64 out of 90 (21 of 27) individuals who explained the game were male. The results are significant at p<0.001 (p = 0.016). S6 Table summarizes these results.

#### Analysis of facial expressions

In contrast to content analysis, analysis of facial expressions was done using machine learning (random forest). We did not apply any coders. Instead, the extraction of facial features was done automatically using OpenFace [56]. The precise technical approach is codeveloped by the author and is illustrated in [13].

To predict the contribution rates in the last period, a binary classifier was trained to predict whether all subjects of a group will contribute fully in the last period of the experiment. The dataset consists of 127 different groups divided into 24 experimental sessions. Given the experimental design, the same subject might appear at most in two groups (in communication in blocks two and three), but only within the same session. Therefore, using leave-one-session-out cross-validation enables training of person-independent models for the analysis. This ensures that no subject appears in the training and test set simultaneously.

The results show that predicting group behavior based on facial visual cues from the FFC video is complex, only slightly better than the trivial model, but feasible. The predictions are better than guessing. Since guessing is defined as 50% we do not consider this as a valuable benchmark. Instead, we focus on an informed guess, which means the decision is always in favor of the majority class (i.e., full contribution). This, we refer to as a trivial model. We compare the accuracy rates (central criterion from engineering perspective) of these models with those focusing on the first half, second half, and the complete video. This task was expected to be especially difficult since the decisions are subject to much more hidden influences and data quality was not optimal. Nevertheless, on average, end models predict about 70% of the decisions, which is significantly more than guessing or trivial model (S7 Table).

While our results indicate that it can be possible to predict cooperation only by analyzing facial expressions, we acknowledge another intriguing question: which expressions are associated with greater cooperation? Given the applied methodology, this question cannot be answered completely. Yet, in Table 9, we provide an overview of the feature importance of head posture and different action units (AU) distinguishing between the presence of the action unit and its intensity. Here, feature importance is a score indicating the importance of the feature for the prediction model. An action unit refers to a simple action in the face, e.g. AU01 refers to raising the inner brow. The presence measures whether it can be detected and intensity measures in how far there was only a trace of the AU (raising the brow a little) or it was at its maximum value. A more detailed analysis of the action units can be found in [13]. The Table shows that the posture of the head receives the highest feature importance, followed by the intensity features and presence features. While these features can be regarded as indications of what expressions relate to higher cooperation, there are two important limitations. First, these values are technical ones and do not imply that head posture predicts free-riding. The depicted features are likely to occur interdependently, which limits the interpretation of individual features taken out of the context of the random forest classification. Second, even assuming feature importance is used to identify most important facial expressions, it still only applies to computer vision.

**Table 9:** Features importance for the FF4 by Using RFc (5k).

Imp	Feature	Value	Imp	Feature	Value	Imp	Feature	Value	Imp	Feature	Value
1	pose_y	0.1387	7	AU17_I	0.1029	13	AU07_I	0.0759	19	AU26_P	0.0696
2	pose_p	0.1367	8	AU02_I	0.1022	14	AU20_P	0.0744	20	AU09_P	0.0667
3	pose_r	0.1349	9	AU15_I	0.1012	15	AU45_P	0.0736	21	AU01_P	0.0640
4	AU45_I	0.1060	10	AU23_P	0.0769	16	AU02_P	0.0721			
5	AU20_I	0.1036	11	AU05_P	0.0766	17	AU28_P	0.0717			
6	AU23_I	0.1033	12	AU14_I	0.0763	18	AU04_P	0.0707			

Obtained from [13]. The features in the table ordered by their importance from most important to least important. I—intensity, P—presence.

## Discussion

The paper presented several findings. Before discussing these findings separately, it is important to address some methodological concerns. Parts of the analysis rely on small observation numbers. This is mainly due to the endogenous decision process. Thus, it was a priori difficult to precisely estimate the numbers of groups that will fund the institutions. Still, the measured effect size is a small (0.296) and would require sample sizes of 149 groups per treatment to investigate at the power of 0.8. However, the issue of sample size does not concern the main findings on the reverberation effect but only findings on the signals caused by the formation of the institution. Further, note that for the application of machine learning, a higher number of observations would be beneficial. This refers to the idea that to obtain better machine learning results, it would be better to operate with much more than 127 videos. However, making decisions on sample size based on this criterion would require a very high number of participants. This is impractical. Further, in such a case, even small effects may become significant at conventional levels of significance.

Concerning signals, the paper did not find signaling effects of the (un)successful funding of the communication platform. The differences in contribution behavior between having the option to fund the institution or being allocated to the respective scenario are small and not significant. It is noteworthy that the potential effect is a priori limited for groups with C-VCM in block three. Since contribution rates in block two were very high the only measurable effect could have been decreasing rates. Yet, this was not observed. However, the result from subjects without communication in block three are more ambiguous. A priori it was reasonable to assume that not being able to fund communication may send out a trust signal. Yet, as trust and regulation can operate bi-directionally [44, 45], it was not a priori clear whether the signal would be positive or negative. Experimental data indicates contribution rates in NF were consistently higher than in S throughout all ten periods of block three. However, the effect size was small (0.296) and differences were insignificant. To conclude, no significant differences concerning funding communication were found. This can be regarded in a minor contrast to the “endogeneity premium” [57]. However, in the case of this experiment, the formation was costly, and the motives in favor or against repeating communication potentially differed among subjects due to different experiences.

Concerning the reverberation effect which is observed in a repeated VCM structure, it is worth recalling the results from [8–10] on restarts with strangers. There, the authors repeated the VCM procedure including randomizing the group members between the retakes, as it was done in the present paper. The authors observed that after every restart the contributions went up, yet remained lower than the initial contributions in the first block. Furthermore, the contributions followed a similar pattern after the restart. In our experiment, subjects who repeated the VCM in block three had higher initial contributions and had a slightly different contribution pattern over time. Note that in the experiment there was no exogenous variation of communication in block two. One solution to it, is to implement a treatment where subjects repeat the VCM three times and compare contributions in block three of such a treatment to those in NF and S. However, given the vast amount of literature indicating a clear path how contributions decrease over time [8–12, 43, 52, 58], it is clear that contributions in block three of such a treatment will be no higher than in block one. This means that testing average contributions of block one versus block three as in this article can only lead to underestimating the true effect size, despite the difference being already highly significant (p<0.0001).

Since between block three and one there was nothing else but block two, and the experiment made use of random incentive systems to avoid income accumulation, the higher contributions rates in block three may only stem from block two which included communication. However, as always with initial experimental findings, these explanations need further empirical support, dealing with the question which elements of communication relate to the lasting effect of communication. This relates to the research questions raised in [22]. One suitable explanation is that the combination of the inefficient VCM and the efficient C-VCM led to a more frequent emergence of a tit-for-tat type of strategy as described in [59] or [60]. The combined experiences of the VCM in the first and C-VCM in the second block potentially induced two observations among subjects. Firstly, it is financially beneficial to have high and stable group contributions. Secondly, once members of the group start free-riding it is evident that the cooperation will break down. To avoid exploitation by free-riders, individuals adapted by faster reducing their contributions in block three. These two observations combined may be the explanation for the illustrated reverberation effect of communication. Further, these findings are in line with the learning theory of end-game behavior [16] and the research on multiple games environment as in [61]. The latter illustrated learning spillover effects and demonstrated how subjects learn to behave in strategically equivalent games in the same way. Yet, it is also worth noting that these findings are in slight contrast to [42] who did not find such an effect. However, the experiment differs in terms of how the subjects experienced the difference between the efficient and inefficient VCM and the type of intervention. The interventions did not achieve the high contribution rates that were obtained in this research due to pre-play communication and eventually backfired once removed. A further limitation is that we cannot address whether the effect would occur when subjects are replaced periodically as in [21].

Another line of argument stems from evolutionary game theory, according to which cooperation required to solve social dilemmas can evolve from five mechanisms: kin selection, direct/indirect/network reciprocity, and group selection [62]. In this line of thinking communication can contribute to such a social viscosity by, e.g. authenticating others [63]. Thus, it is possible that subjects in the experiment, by the means of communication, verify similarities of the common kin and therefore increase their reciprocal contributions. However, such an effect is difficult to isolate experimentally, as the best approach would be to implement computerized players as discussed in the review on economic experiments using bots [64].

Concerning the analysis of communication, we aim to discuss a combination of both approaches. Content analysis indicates that groups that communicated more, were more likely to have full contributions in the last period. Analysis of facial expressions indicates that the last half of communication is more informative. There is not enough information to analyze whether these findings are interconnected. We consider two plausible reasons for why they may be. First, as face-to-face communication is shown to effectively increase contributions, the duration of communication can be an influencing factor, too. Groups that stop talking to each other and look around instead, have fewer words spoken and the algorithm can theoretically spot people looking in a different direction than the screen. Second, the most important content topic was discussing the end-game. This topic cannot be raised at the beginning of the discussion. Instead, it can only come up after somebody explained the game or the group discussed that full contributions are the socially optimal choice. This means, that it is more likely that the topic was raised in the second half of the communication. Still, this remains speculative as joint analysis of content and the respective facial expressions was not possible due to technical limitations. Further, some of our results indicate differences between FTC and STC, implying that learning did not only happen on the contribution but also on communication level. However, this requires more research.

## Conclusion

The focus of the research was to analyze whether positive effects of face-to-face communication through ICT prevail after its removal and how individuals react to the failure to finance the renewal of the communication platform. The analysis leads to several conclusions. Firstly, there is strong evidence that communication affected the contribution behavior even after it was removed—despite the groups being randomized. The positive experiences gathered in C-VCM affected contributions in the subsequent VCM. However, the individuals still experienced the end-game effect. Compared to VCM block one, in the VCM in block three contributions were higher but experienced a steeper decrease at the end of the game. As briefly discussed, this may be due to overlapping learning processes in different environments. This type of learning may be the main driver of the reverberation effect of communication after its removal. Repeating communication did not have any significant effects. This is foremost because the contribution rates were already at an extraordinarily high level. To conclude, the experiment illustrated that subjects were able to learn simultaneously from positive and negative experiences of the theoretically identical mechanisms.

Further, the paper provides evidence that the way communication is paid for is less important. The most important factor is whether communication took place. Still, the results illustrate that communication, being highly efficient, loses its beneficial effects over time. The implications of this paper are twofold. On the one hand, it illustrates that even after removing communication, the positive effects remain to a certain degree. On the other hand, the paper stresses the possibility of this effect reverberating and eventually fading away completely if no measures are taken to solve the initial dilemma. Consequently, long-term path dependence based on the quality of the institution was not observed. However, the discussion is limited to pre-play face-to-face communication and no implications are made on formal institutions (e.g. punishing free-riders) since these often operate on a different channel.

## Supporting information

S1 File Translated instructions of the experiment. (PDF)

S1 Table Differences between refund and no-refund treatments in block three aggregated on the group level over 10 periods and p-values of the MW-test. (PDF)

S2 Table Mistrust effect after the failed funding of communication. (PDF)

S3 Table Panel Tobit regression results on group and individual level for individuals without communication (NF and S) in the third block. (PDF)

S4 Table Panel tobit regression results on group and individual level for individuals with communication (FC and C) in the third block. (PDF)

S5 Table Interrater agreement. (PDF)

S6 Table Analysis of communication leadership concerning gender and economic education. (PDF)

S7 Table MW-Test for comparing the results of uniformed guess, trivial models, combined models, beginning models and end models. (PDF)
