# Supplementary material for: Reverberation effect of communication in a public goods game
Source: PLoS One. 2023 Feb 27;18(2):e0281633. doi: 10.1371/journal.pone.0281633 (PMC9970058; doi:10.1371/journal.pone.0281633)
Supplement: S1 File — (PDF) [file pone.0281633.s001.pdf]

Translated instructions of the experiment

Instructions Experiment “Yellow“

Please read the instructions diligently. If questions arise, open the door to your cabin and remain seated. The experiment “Yellow” is carried out at the computer.
Your fellow participants will only play with you within the experiment “Yellow“.

After reading the instructions you will receive four control questions. The control questions are not considered for your final payment. As soon as you have answered the control questions the part of the experiment relevant for your final payment will start. Please be aware that either experiment „Yellow“, „Blue“ or „Red“ will be paid out. Which experiment will be eventually relevant is decided by chance.

Within the experiment, we will use laboratory dollars as the used currency. The underlying exchange rate is the following: 100 laboratory dollars = 4.5 EUR.

You and three other participants receive each **20 laboratory dollars** per round of contribution. You can contribute these laboratory dollars either to a private or a group account.

Private Account (P): The deposited laboratory dollars are being kept.

Group Account (G): Each of the four players can deposit money in this account. The sum of the deposits is doubled by the experimenter and redistributed equally to the four players. Hence, each player receives 0,5 laboratory dollars per contributed laboratory dollar.

The laboratory dollars can be split up in between the two accounts. You take your decision anonymously. None of the other players will learn how you split your laboratory dollars up. Profit of player i is calculated accordingly:

$$\text{Profit}=(20-G)+0{,}5\cdot\sum_{1}^{4} G_i$$

Please turn!

Instructions Experiment „Red"

Please read the instructions diligently. If questions arise, open the door to your cabin and remain seated. The experiment "Red" is carried out at the computer.
Your fellow participants will only play with you within the experiment „Red".

After reading the instructions you will receive four control questions. The control questions are not considered for your final payment. As soon as you have answered the control questions the part of the experiment relevant for your final payment will start. Please be aware that either experiment „Yellow", „Blue" or „Red" will be paid out. Which experiment will be eventually relevant is decided by chance.

Within the experiment, we will use laboratory dollars as the used currency. The underlying exchange rate is the following: 100 laboratory dollars = 4.5 EUR.

You and three other participants receive each **20 laboratory dollars** per round of contribution. You can contribute these laboratory dollars either to a private or a group account.

Private Account (P): The deposited laboratory dollars are being kept.

Group Account (G): Each of the four players can deposit money in this account. The sum of the deposits is doubled by the experimenter and redistributed equally to the four players. Hence, each player receives 0,5 laboratory dollars per contributed laboratory dollar.

The laboratory dollars can be split up in between the two accounts. You take your decision anonymously. None of the other players will learn how you split your laboratory dollars up. Profit of player i is calculated accordingly:

$$\text{Profit}=(20-G)+0,5\cdot\sum_{1}^{4} G_i$$

Video conference: Before you take your decision on how to split the laboratory dollars you will be talking to the three other players in a video conference for three minutes. During this time, you can see and talk to each other. The duration of the call can neither be reduced nor prolonged.
Subsequently to the video conference, each player makes the above described decision.

Please turn!

Instructions Experiment „Blue" (Without Refund)

Please read the instructions diligently. If questions arise, open the door to your cabin and remain seated. The experiment "Blue" is carried out at the computer.
Your fellow participants will only play with you within the experiment „Blue".

After reading the instructions you will receive six control questions. The control questions are not considered for your final payment. As soon as you have answered the control questions the part of the experiment relevant for your final payment will start. Please be aware that either experiment „Yellow", „Blue" or „Red" will be paid out. Which experiment will be eventually relevant is decided by chance.

Within the experiment, we will use laboratory dollars as the used currency. The underlying exchange rate is the following: 100 laboratory dollars = 4.5 EUR.

You and three other participants receive each **20 laboratory dollars** per round of contribution. You can contribute these laboratory dollars either to a private or a group account.

Private Account (P): The deposited laboratory dollars are being kept.

Group Account (G): Each of the four players can deposit money in this account. The sum of the deposits is doubled by the experimenter and redistributed equally to the four players. Hence, each player receives 0,5 laboratory dollars per contributed laboratory dollar.

The laboratory dollars can be split up in between the two accounts. You take your decision anonymously. None of the other players will learn how you split your laboratory dollars up. Profit of player i is calculated accordingly:

$$\text{Profit}=(20\text{-}G)+ 0{,}5\cdot \sum_{1}^{4} G_i$$

Set-up of the video conference: At the beginning of the experiment you are asked whether you want to make the experiment this time with or without communication. Communication will be subject to a fee. To make the experiment with communication you must raise a required amount jointly as a group. This amount will pop up on your screen at the beginning of the experiment. The decision on how much you contribute will then be again taken anonymously. The deposited money for setting up communication is being deducted from your profit in the experiment „blue" at the end of it – whether communication is successfully set up or not. If the group raises the required amount, a three-minute video conference is being set up, see previous round. Otherwise, all group members have to wait for three minutes until other groups have finished their communication period, respectively. Subsequently, the decision on how to split up the laboratory dollars between private and group account are being made.

Please turn!

Instructions Experiment „Blue" (With Refund)

Please read the instructions diligently. If questions arise, open the door to your cabin and remain seated. The experiment "Blue" is carried out at the computer.
Your fellow participants will only play with you within the experiment „Blue".

After reading the instructions you will receive six control questions. The control questions are not considered for your final payment. As soon as you have answered the control questions the part of the experiment relevant for your final payment will start. Please be aware that either experiment „Yellow", „Blue" or „Red" will be paid out. Which experiment will be eventually relevant is decided by chance.

Within the experiment, we will use laboratory dollars as the used currency. The underlying exchange rate is the following: 100 laboratory dollars = 4.5 EUR.

You and three other participants receive each **20 laboratory dollars** per round of contribution. You can contribute these laboratory dollars either to a private or a group account.

Private Account (P): The deposited laboratory dollars are being kept.

Group Account (G): Each of the four players can deposit money in this account. The sum of the deposits is doubled by the experimenter and redistributed equally to the four players. Hence, each player receives 0,5 laboratory dollars per contributed laboratory dollar.

The laboratory dollars can be split up in between the two accounts. You take your decision anonymously. None of the other players will learn how you split your laboratory dollars up. Profit of player i is calculated accordingly:

$$\text{Profit}=(20\text{-}G)+ 0{,}5\cdot \sum_{1}^{4} G_i$$

Set-up of the video conference: At the beginning of the experiment you are asked whether you want to make the experiment this time with or without communication. Communication will be subject to a fee. To make the experiment with communication you must raise a required amount jointly as a group. This amount will pop up on your screen at the beginning of the experiment. The decision on how much you contribute will then be again taken anonymously. The deposited money for setting up communication is being deducted from your profit in the experiment „blue" at the end of it – only if communication is successfully set up. If the group raises the required amount, a three-minute video conference is being set up, see previous round. Otherwise, all group members have to wait for three minutes until other groups have finished their communication period, respectively. Subsequently, the decision on how to split up the laboratory dollars between private and group account are being made.

Please turn!
