# Supplementary material for: Reverberation effect of communication in a public goods game
Source: PLoS One. 2023 Feb 27;18(2):e0281633. doi: 10.1371/journal.pone.0281633 (PMC9970058; doi:10.1371/journal.pone.0281633)
Supplement: S1 Table — (PDF) [file pone.0281633.s002.pdf]

Table S1 Differences between refund and no-refund treatments in Block Three aggregated on the Group Level over 10 periods and p-values of the MW-test.

	FCnr	FCr	NFnr	NFr
Mean	790.29	777.50	648.00	637.58
Observations	7	8	25	24
MW-test	0.4490		0.7793	

Note: As the results are conducted on the group level there is only a limited number of observations, especially in the case of FC (15 observations). Therefore, the results need to be considered with caution.
