# Supplementary material for: Reverberation effect of communication in a public goods game
Source: PLoS One. 2023 Feb 27;18(2):e0281633. doi: 10.1371/journal.pone.0281633 (PMC9970058; doi:10.1371/journal.pone.0281633)
Supplement: S2 Table — (PDF) [file pone.0281633.s003.pdf]

Table S2 Mistrust Effect after the failed funding of communication

	(1)<br>NF	(2)<br>+ Controls	(3)<br>NFr	(4)<br>+ Controls	(5)<br>NFnr	(6)<br>+ Controls
Investment	-1.222**<br>(0.537)	-1.264**<br>(0.535)	-0.385<br>(0.657)	-0.363<br>(0.650)	-2.264**<br>(0.965)	-2.349**<br>(0.993)
FCB1	2.009***<br>(0.405)	1.982***<br>(0.400)	1.777***<br>(0.479)	1.779**<br>(0.472)	2.435***<br>(0.729)	2.401***<br>(0.729)
FCB2	3.278**<br>(1.660)	3.191*<br>(1.662)	2.724*<br>(1.545)	2.705*<br>(1.551)	(omitted)	(omitted)
InstBen	0.009<br>(0.040)	0.008<br>(0.040)	-0.016<br>(0.048)	-0.018<br>(0.047)	0.029<br>(0.071)	0.024<br>(0.071)
Treatment	0.665<br>(1.517)	0.455<br>(1.512)				
Constant	-51.807<br>(33.699)	-38.694<br>(35.428)	-40.001<br>(29.998)	-25.195<br>(32.399)	15.315**<br>(7.664)	27.084<br>(30.802)
Controls	N	Y	N	Y	N	Y
Observa-tions	196	196	96	96	100	100

Note: Standard error is denoted in brackets. ***/**/* denote significance at 0.01/0.05/0.1 levels respectively. Control variables include: gender, age, and study program. The coefficients of FCB2 in the no refund treatment are omitted statistically due to the lack of any variance (every subject contributed 20 LD).[1]

[1] Results are obtained from a Tobit regression model: $FCB3_i = \beta_0 + \beta_1 INV_i + \beta_2 FCB1_i + \beta_2 FCB2_i + \beta_4 InstBen_i + \alpha_i + \varepsilon_i$. Here, FCBi is the first contribution in block i, INV the investment into the communication platform, InstBen the benefit of having the communication (individual differences in payoffs between the second and first block), α set of control variables for the individual i.

Analyzing the first period of block three of those groups that did not successfully fund the institution independent of the refund option, it becomes apparent that high contributions to the communication platform hurt the first contributions. This is depicted in columns (1) and (2). In the treatment with a refund (3) and (4), the effect of high but futile contribution to the communication platform on the first contributions in block three is negligible. This is reasonable since no subject factually lost their investment. However, without the refund option contributions are negatively affected by investment in communication (5) and (6). Yet, this effect is strongly limited in time. Starting from the second contribution period investments in the communication do not matter. Instead, the individual contributions depended more on the contributions of their co-players in the actual VCM as is shown in table A3.
