# Supplementary material for: Reverberation effect of communication in a public goods game
Source: PLoS One. 2023 Feb 27;18(2):e0281633. doi: 10.1371/journal.pone.0281633 (PMC9970058; doi:10.1371/journal.pone.0281633)
Supplement: S3 Table — (PDF) [file pone.0281633.s004.pdf]

Table S3 Panel Tobit Regression Results on Group and Individual Level for Individuals *Without* Communication (NF and S) in the Third Block

Dep. Variable: Contributions	(1) Basis Individual	(2) Interaction Individual	(3) + Control	(4) Basis Group	(5) Interaction Group	(6) + Control
Period	-3.060*** (0.173)	-1.889*** (0.218)	-1.889*** (0.219)	-4.704*** (0.275)	-3.745*** (0.393)	-3.739*** (0.388)
Block = 2	29.737*** (1.594)	41.875 *** (4.232)	41.837 *** (4.229)	69.273*** (3.375)	88.345*** (7.020)	89.313*** (7.076)
Block = 3	7.950*** (0.9574)	24.358*** (2.421)	24.378*** (2.422)	22.897*** (2.707)	31.452*** (4.324)	32.697*** (4.330)
Block (=2) x Period		-1.927*** (0.538)	-1.923*** (0.538)		-2.923*** (0.881)	-2.935*** (0.878)
Block (=3) x Period		-2.658*** (0.350)	-2.659*** (0.350)		-1.516** (0.592)	-1.551*** (0.586)
AvContr Co-players (-1)	0.941*** (0.036)	0.956*** (0.0363)	0.956*** (0.036)			
Constant	-0.922 (1.932)	-8.485*** (2.189)	-13.762* (7.879)	80.535*** (3.217)	75.332*** (3.573)	131.841*** (21.476)
Controls	N	N	Y	N	N	Y
Observations	7020	7020	7020	1950	1950	1950

Note: Standard error is denoted in brackets. ***/**/* denote significance at 0.01/0.05/0.1 levels respectively. Control variables include gender, age, and study program on an individual level or aggregates of these on the group level respectively. For the coefficients obtained for the variable block, the first block was taken as the reference.
