# Supplementary material for: Reverberation effect of communication in a public goods game
Source: PLoS One. 2023 Feb 27;18(2):e0281633. doi: 10.1371/journal.pone.0281633 (PMC9970058; doi:10.1371/journal.pone.0281633)
Supplement: S4 Table — (PDF) [file pone.0281633.s005.pdf]

Table S4 Panel Tobit Regression Results on Group and Individual Level for Individuals *With* Communication (FC and C) in the Third Block

Dep. Variable: Contributions	(1) Basis Individual	(2) Interaction Individual	(3) +Control	(4) Basis Group	(5) Interaction Group	(6) + Control
Period	-2.703***	-7.763***	-7.766***	-4.193***	-6.760***	-7.180***
	(0.257)	(1.261)	(1.260)	(0.351)	(1.139)	(1.211)
Block = 1	-29.990***	-76.430***	-76.446***	-69.287***	-91.448***	-96.849***
	(2.361)	(11.477)	(11.465)	(4.713)	(10.162)	(10.995)
Block = 3	3.058	34.264	34.446	1.927	-6.482	-8.748
	(2.321)	(21.197)	(21.193)	(3.595)	(12.127)	(12.745)
Block (=1) x Period		5.948***	5.946***		3.146***	3.571***
		(1.275)	(1.273)		(1.203)	(1.270)
Block (=3) x Period		-3.323	-3.343		1.129	1.539
		(2.294)	(2.294)		(1.524)	(1.579)
AvgContr (t-1)	0.639***	0.658***	0.657***			
	(0.055)	(0.054)	(0.054)			
Constant	39.862***	80.069***	70.858***	144.046***	163.443***	80.000***
	(4.744)	(11.691)	(14.660)	(5.401)	(10.127)	(29.520)
Controls	N	N	Y	N	N	Y
Observations	3348	3348	3348	930	930	930

Note: Standard error is denoted in brackets. ***/**/* denote significance at 0.01/0.05/0.1 levels respectively. Control variables include: gender, age, and study program on an individual level or aggregated on the group level respectively. For the coefficients obtained for the variable block, the second block was taken as the reference.
