# Supplementary material for: Reverberation effect of communication in a public goods game
Source: PLoS One. 2023 Feb 27;18(2):e0281633. doi: 10.1371/journal.pone.0281633 (PMC9970058; doi:10.1371/journal.pone.0281633)
Supplement: S5 Table — (PDF) [file pone.0281633.s006.pdf]

Table S5 Interrater Agreement

	Full Invest-ment	End-game awareness	Previous expe-rience	Threat & conse-quences	Disagree-ment	Informati on provider
Percent Agreement	1.0000	0.8730	0.8810	0.9048	0.9841	0.9508
Krippendorff's Alpha	1.0000	0.7187	0.7628	0.7748	0.9008	0.9473
Brennan and Prediger	1.0000	0.7460	0.7619	0.8095	0.9683	0.9477
Cohen/Conger's Kappa	1.0000	0.7181	0.7635	0.7749	0.9000	0.9471
Scott/Fleiss' Pi	1.0000	0.7165	0.7619	0.7729	0.8997	0.9472
Gwet's AC	1.0000	0.7700	0.7619	0.8360	0.9811	0.9478
Number of raters	2	2	2	2	2	2
Ratings per rater	127	127	127	127	127	127
