# Supplementary material for: Reverberation effect of communication in a public goods game
Source: PLoS One. 2023 Feb 27;18(2):e0281633. doi: 10.1371/journal.pone.0281633 (PMC9970058; doi:10.1371/journal.pone.0281633)
Supplement: S6 Table — (PDF) [file pone.0281633.s007.pdf]

Table S6 Analysis of communication leadership concerning gender and economic education.

		Female	Male		Economist	Non-Economist	
FTC	**Talker**						
	No	147	139		153	133	
	Yes	25	73		49	49	
	Chi2			*0.000*			*0.550*
	**Information provider**						
	Yes	138	136		143	132	
	No	26	64		49	41	
	Chi2			*0.000*			*0.666*
STC	**Talker**						
	No	49	44		50	43	
	Yes	6	25		13	18	
	Chi2			*0.001*			*0.254*
	**Information provider**						
	No	43	46		41	48	
	Yes	6	21		18	9	
	Chi2			*0.016*			*0.061*

Note: p-values come from Chi2 tests. Results are based on 384 individuals in FTC and 124 individuals in STC. Individuals who had the highest word count in the group are coded as “talkers”. Individuals who explained the game are coded as “information providers”. Cases where coders disagreed are left out.
