# Supplementary material for: Reverberation effect of communication in a public goods game
Source: PLoS One. 2023 Feb 27;18(2):e0281633. doi: 10.1371/journal.pone.0281633 (PMC9970058; doi:10.1371/journal.pone.0281633)
Supplement: S7 Table — (PDF) [file pone.0281633.s008.pdf]

Table S7 MW-Test for comparing the results of uniformed guess, trivial models, combined models, beginning models and end models.

Models	p-value
uninformed guess & trivial models	0.0000
uninformed guess & combined models	0.0000
uninformed guess & beginning models	0.0000
uninformed guess & end models	0.0000
trivial models & combined models	0.0530
trivial models & beginning models	0.0519
trivial models & end models	0.0082
combined models & beginning models	0.9872
combined models & end models	0.4435
beginning models & end models	0.5147

Note: Obtained from [13]. The tests were applied on the respective accuracy rates coming from the leave-one-session-out cross-validation. The rank of these models from the best to worst based on p-value is the end models, the beginning models, combined models, trivial models, and uninformed guess respectively.
